# Synthesis of nano-sized tungsten oxide particles encapsulated in a hollow silica sphere and their photocatalytic properties for decomposition of acetic acid using Pt as a co-catalyst†

**DOI:** 10.1039/d0ra01988g

**Published:** 2020-04-17

**Authors:** Takashi Harada, En Yagi, Shigeru Ikeda

**Affiliations:** Research Center for Solar Energy Chemistry, Osaka University 1-3 Machikaneyama Toyonaka 560-8531 Japan harada@chem.es.osaka-u.ac.jp; Department of Chemistry, Faculty of Science and Engineering, Konan University Okamoto, Higashinada Kobe 658-8501 Japan

## Abstract

Nano-sized tungsten oxide (WO_3_) particles, each of which was encapsulated as a core in a hollow silica sphere (WO_3_@SiO_2_), were synthesized using calcium tungstate particles as the starting material. The calcium tungstate particles, each of which was covered with a silica shell, were converted to tungstic acid by nitric acid treatment and then to WO_3_ by heat treatment to obtain WO_3_@SiO_2_. A hollow space was formed in WO_3_@SiO_2_ between the WO_3_ core and the SiO_2_ shell as a result of shrinkage of WO_3_ during the heat treatment. The thus-obtained WO_3_@SiO_2_ was 40 nm in diameter, the WO_3_ core was 10 nm in diameter, and the silica shell, which was permeable to gas and liquid, was 10 nm in thickness. WO_3_@SiO_2_ absorbed visible light to the wavelength of 454 nm, which enabled photocatalytic reaction under visible light; Pt was loaded on the WO_3_ cores in the photocatalytic reactions. In contrast to Pt-loaded bulk WO_3_ photocatalysts without an SiO_2_ shell, Pt-loaded WO_3_@SiO_2_ showed continuous and complete decomposition of gaseous acetic acid in air under visible as well as UV irradiation.

## Abstract 2

We present a procedure for the synthesis of WO_3_ nanoparticles encapsulated in a hollow silica sphere, and their unique photocatalytic properties.

## Graphical abstract

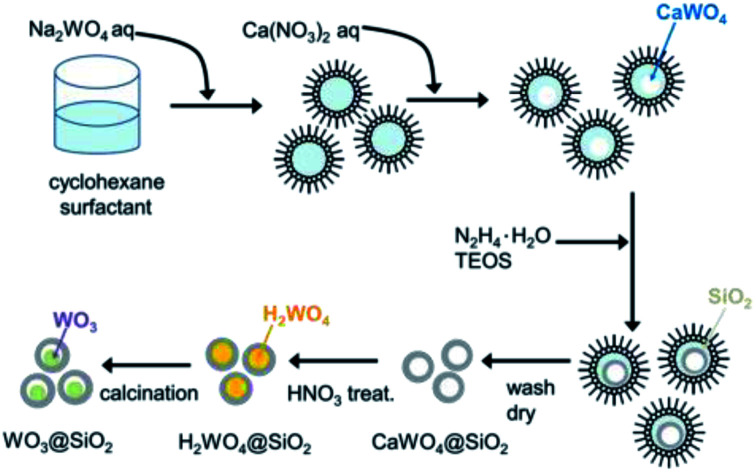

## Introduction

Titanium dioxide (TiO_2_) is well known as the most efficient photocatalyst and has been used in various applications, such as production of renewable energy, decomposition of environmental pollutants and synthesis of useful organic compounds, because electrons and holes photogenerated in TiO_2_ have strong reduction and oxidation properties, respectively.^1–3^ However, due to its strong oxidation property, TiO_2_ indiscriminately decomposes organic compounds, which sometimes leads to problems of decomposition of the support of TiO_2_ photocatalyts. To solve this problem, a core–shell structure in which TiO_2_ particles are covered with robust inorganic materials has been proposed.^4^ The shell is permeable to gas and liquid, while it prevents contact between TiO_2_ particles, leading to suppression of degradation of supports made of organic materials. However, the structure had the disadvantage of lowered photocatalytic activity because a large part of the TiO_2_ surface, which is important for the photocatalyst, was covered with the shell material. To solve the problem, we have recently developed a TiO_2_ photocatalyst encapsulated in a hollow silica shell (TiO_2_@SiO_2_), in which a gap space is formed between the core and the shell. As a result, the TiO_2_@SiO_2_ showed photocatalytic activity as high as that of an intrinsic TiO_2_ photocatalyst, and showed size-selectivity for materials to be decomposed, depending on the pore size of the permeable shell.^5–8^ In addition, other features of core–hollow shell structure, such as improvement adsorption properties, high temperature stability of core material and application for cascade reaction, have been reported.^9–11^

There have been many efforts to modify TiO_2_ so as to enable absorption of visible light; an intrinsic TiO_2_ photocatalyst shows low photocatalytic activity in normal environments because it shows photocatalytic activity only under UV light due to its large band gap (3.2 eV). Approaches to solve this problem include doping of TiO_2_ with transition metals^12–15^ and replacement of oxygen in TiO_2_ with sulfur or nitrogen.^16–21^ Another approach is to use low band gap materials instead of TiO_2_. Tungsten oxide (WO_3_) is a good candidate of these materials because of its narrow band gap (2.8 eV), high oxidation property, nontoxicity, and chemical stability.^22^ However, a pure WO_3_ photocatalyst shows low photocatalytic activity for decomposition of organic compounds because of the low energy level of its conduction band (CB) edge, which is not high enough for the electrons in the CB to reduce O_2_ or H_2_O. Sayama *et al.* reported that loading co-catalysts, such as CuO, Pd and Pt, on the surface of WO_3_ was effective for improving its photocatalytic activity.^23,24^ Although the co-catalysts do not affect the energy level of the CB of WO_3_, they kinetically enhance the electron transfer from WO_3_ to O_2_ or H_2_O and, as a result, increase photocatalytic activity of WO_3_. We expect that WO_3_ particles encapsulated in a hollow silica sphere (WO_3_@SiO_2_) would be suitable as a photocatalyst used under visible light.

A number of synthesis procedures have been developed to produce the core–hollow shell structure using, for example, the Kirkendall effect,^25^ a hard template,^26^ and an etching process.^27^ We previously reported the synthesis of TiO_2_@SiO_2_ using a carbon material as a hard template.^5–8^ The hard template method is useful for obtaining well-defined core–hollow shell structures.^5–8,26^ However, this method requires the formation and removal of solid templates, which are troublesome procedures, especially for practical use. Moreover, core–hollow shell structures thus-obtained are often large in size and irregular in shape. Because these structures show low mechanical stability due to the large hollow space, the structure needs to be small and, ideally, spherical.

We recently developed a simple procedure for making nickel nanoparticles encapsulated in a hollow silica sphere.^28^ In this procedure, small crystals of a nickel hydrazine complex were prepared in a reverse microemulsion system and were directly covered with a silica layer. The nickel hydrazine crystals were converted to nickel nanoparticles by heat treatment. Because the nickel nanoparticles were smaller than nickel hydrazine crystals, a core–hollow shell structure was automatically obtained during the heat treatment. Here, we applied this strategy to the synthesis of WO_3_@SiO_2_ and evaluated its photocatalytic activity for decomposition of acetic acid in gas phase, which was used as a model reaction.

## Experimental

### Synthesis of tungsten oxide photocatalysts

Tungsten oxide encapsulated in a hollow silica sphere (WO_3_@SiO_2_) was synthesized as follows. To 34 g polyoxyethylene cetyl ether (*n* = 15) (30 mmol) dissolved in 75 cm^3^ of cyclohexane were added 4.5 cm^3^ of 0.1 M aqueous Ca(NO_3_)_2_ (0.45 mmol) and 4.5 cm^3^ of 0.1 M aqueous Na_2_WO_4_ (0.45 mmol). After the mixture had been stirred at 55 °C for 1 h, 1.5 cm^3^ of hydrazine monohydrate (30 mmol) was added and the mixture was stirred at 55 °C for 1 h. Then 3 cm^3^ of tetraethylorthosilicate (TEOS: 13.4 mmol) was added to the suspension, which was stirred at 55 °C for 2 h. After addition of 240 cm^3^ of 2-propanol to the suspension, the mixture was centrifuged (3000 rpm, 4 min) to recover the precipitate, which was washed with 2-propanol three times. The precipitate was added to 2.5 M aqueous HNO_3_ at 80 °C for 6 h and then washed with distilled water until the effluent showed neutral pH. After drying at 80 °C, the powder was calcined at 700 °C for 2 h in air to produce WO_3_@SiO_2_.

As a reference photocatalyst, we synthesized bulk WO_3_ (WO_3_(B)) powder by the same procedure but without the use of the reverse micelle system and silica source. WO_3_(B) physically mixed with SiO_2_ (WO_3_(B)/SiO_2_) was also used as a reference photocatalyst. This reference photocatalyst was prepared using H_2_WO_4_, which is a precursor of WO_3_(B), and about 30 nm of SiO_2_ powder, which is synthesized by a slightly modified Stöber method.^29^ They were mechanically mixed and calcined at 700 °C. The WO_3_ content of WO_3_(B)/SiO_2_ was adjusted to be the same as that of WO_3_@SiO_2_.

### Characterization

TEM images were taken on a Hitachi H-9000NAR instrument operated at 300 kV. Powder X-ray diffraction (XRD) patterns were recorded using a Rigaku MiniFlex X-ray diffractometer (CuKα, Ni filter). Analyses of the surface area and pore structure were carried out using a Quantachrome AUTOSORB-1 automated gas-sorption system with N_2_ as the adsorbate, after pretreatment of the sample at 200 °C for 2 h under reduced pressure. The BET surface area was calculated from the adsorption branch of the isotherm. Ultraviolet and visible-light diffuse reflection (UV-vis DR) spectra were obtained with a Hitachi U-4100 UV-vis-NIR spectrometer equipped with an integrating sphere using BaSO_4_ as a reference. The reflectance spectra were converted to absorbance spectra by the Kubelka–Munk method.

### Photocatalytic decomposition of acetic acid in gaseous phase

Photocatalytic decomposition of acetic acid was carried out over Pt-loaded WO_3_ photocatalysts (Pt/WO_3_) in gaseous phase. Pt-loading on WO_3_ photocatalysts was carried out by the photodeposition method sequentially from an aqueous methanol (10 vol%) solution of H_2_PtCl_6_·6H_2_O.^30^ The loading amount of Pt was fixed at 0.5 wt% of WO_3_ for all of the photocatalysts.

Photocatalytic decomposition of acetic acid was performed in a 225 cm^3^ Pyrex cylindrical reaction vessel (64 mm in diameter) with a quartz top window. The photocatalyst, containing 30 mg of WO_3_, was spread on the slide glass (38 mm × 26 mm) and set in the vessel. After closing the vessel, 1 μl of acetic acid (17.5 μmol) was introduced into the vessel. After acetic acid had been fully vaporized and had reached an absorption equilibrium in the vessel, the sample was photoirradiated with a 300 W Xe lamp though the quartz top window. CO_2_ was analyzed with a Shimadzu GC-8A gas chromatograph equipped with a TCD detector and an activated carbon column, and organic compounds were analyzed with a Shimadzu GC-2014 gas chromatograph equipped with a BID detector and a TC-FFAP column. For the analyses, a portion of the gas phase was sampled with an air-tight syringe at appropriate intervals.

## Results and discussion

### Synthesis and characterizations of tungsten oxide nanoparticles encapsulated in a hollow silica sphere

**Fig. 1:**
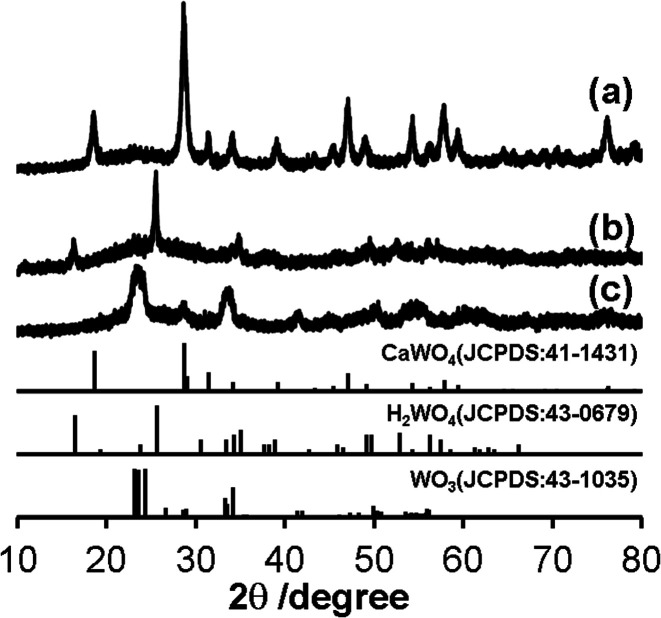
XRD patterns of (a) CaWO_4_@SiO_2_, (b) H_2_WO_4_@SiO_2_ and (c) WO_3_@SiO_2_. The standard data of CaWO_4_ (JCPDS: 41-1431), H_2_WO_4_ (JCPDS: 43-0679) and WO_3_ (JCPDS: 43-1035) are shown as a reference.

**Fig. 2:**
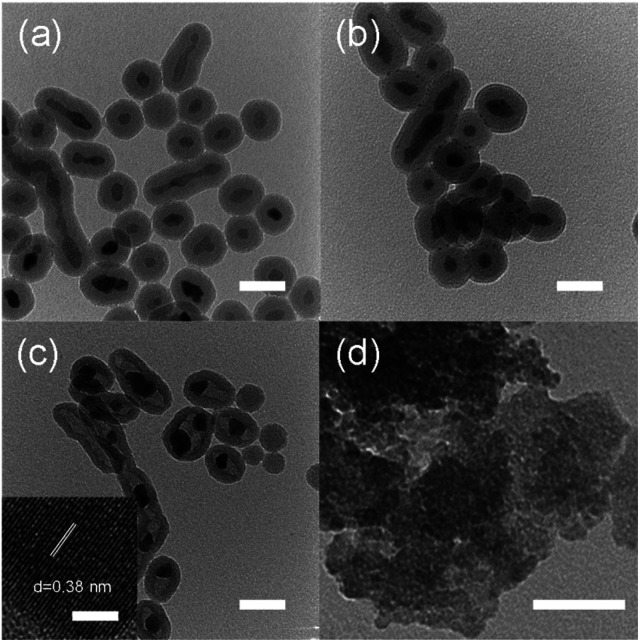
TEM images of (a) CaWO_4_@SiO_2_, (b) H_2_WO_4_@SiO_2_, (c) WO_3_@SiO_2_ and (d) WO_3_(B). Scale bars are corresponding to 40 nm. Inset of (c) is HRTEM image of WO_3_@SiO_2_ and scale bar is corresponding to 5 nm.

Synthesis of nano-sized WO_3_@SiO_2_ was performed in a water in oil (W/O) microemulsion system. In the first step, an aqueous Ca(NO_3_)_2_ solution was added to a cyclohexane solution containing polyoxyethylene cetyl ether (*n* = 15) to form a W/O microemulsion. Addition of an aqueous Na_2_WO_4_ solution into the W/O microemulsion solution induced a color change from transparent to white; it became turbid after a while, indicating formation of solid components. By adding a hydrazine monohydrate solution and TEOS to this solution, polycondensation of the siliceous component was continuously carried out. After 2 h agitation of the mixture, the solid part was collected. Fig. 1a shows an XRD pattern of the thus-obtained white powder. All of the peaks were assigned to CaWO_4_ as referred from the JCPDS card (no. 41-1431). A TEM image of the white powder showed spherical core–shell structure (Fig. 2a). These results indicate that CaWO_4_ particles were directly covered with a silica shell (CaWO_4_@SiO_2_). The diameter of the CaWO_4_ core was 10–20 nm and the thickness of the silica shell was about 10 nm.

The thus-obtained CaWO_4_@SiO_2_ powder was treated with aqueous HNO_3_ solution at 80 °C for 6 h. During HNO_3_ treatment, the white color of the turbid solution gradually changed to yellow. The corresponding XRD pattern of the thus-obtained yellow powder was assigned to H_2_WO_4_ as referred from the JCPDS card (no. 43-0679) (Fig. 1b). The TEM image showed that the core–shell structure was not changed by the HNO_3_ treatment, as shown in Fig. 2b. These results indicate that the CaWO_4_ core particle of CaWO_4_@SiO_2_ was converted to H_2_WO_4_ by the HNO_3_ treatment and that a core–shell material having an H_2_WO_4_ core and a silica shell (H_2_WO_4_@SiO_2_) was obtained.

After calcination of H_2_WO_4_@SiO_2_ at 700 °C, the color changed to light green. The XRD pattern of this sample showed the formation of monoclinic WO_3_ (JCPDS card no. 43-1035) (Fig. 1c), which was obtained by dehydration of H_2_WO_4_. To confirm dehydration of H_2_WO_4_, thermogravimetric-differential thermal analysis (TG-DTA) was carried out. The TG-DTA curves of H_2_WO_4_@SiO_2_ were shown in Fig. S2.† A weight loss with endothermic peak was observed at around 100 °C and 200 °C. These are due to desorption of physically adsorbed water molecule on H_2_WO_4_@SiO_2_ particles and coordinated water molecules of H_2_WO_4_, respectively. A weight loss of exothermic peak was observed at 250–700 °C, due to combustion of surfactant and siliceous compounds including removal of residual coordinated water.^31^ A TEM image and high resolution TEM (HRTEM) image of this sample are shown in Fig. 2c and inset of Fig. 2c, respectively. In contrast to the TEM image of H_2_WO_4_@SiO_2_, a hollow space was observed in this sample, indicating the formation of WO_3_ encapsulated in a hollow silica sphere (WO_3_@SiO_2_). The hollow space was caused by shrinkage of the volume as H_2_WO_4_ was converted to WO_3_. The size of the WO_3_ particles was about 10 nm in diameter, which is smaller than that of the H_2_WO_4_ core in H_2_WO_4_@SiO_2_. The lattice fringes of HRTEM image of WO_3_ particle are assigned to the (100) plane of monoclinic WO_3_. This is agreement with the XRD result. The silica shell thickness seems to be slightly decreased after the heat treatment. A TEM image of WO_3_(B) powder that was prepared without using the reverse microemulsion system and silica source showed that the particle size was large and inhomogeneous (40–100 nm) as can be seen in Fig. 2d, although WO_3_(B) was obtained by the same route as WO_3_@SiO_2_ (Fig. S1†). These results suggest that the use of the reverse microemulsion system and the silica shell structure are effective for suppressing the growth of WO_3_ particles during calcination.

**Fig. 3:**
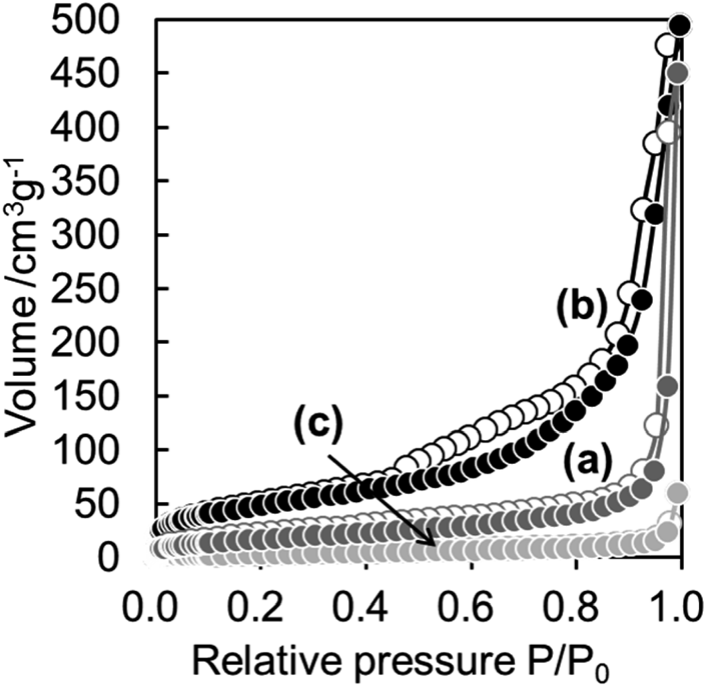
N_2_ sorption isotherms of (a) CaWO_4_@SiO_2_, (b) WO_3_@SiO_2_ and (c) WO_3_(B). Filled and open circles denote adsorption and desorption isotherms, respectively.

Fig. 3 shows N_2_ adsorption–desorption isotherms of WO_3_(B), CaWO_4_@SiO_2_ and WO_3_@SiO_2_ samples. The isotherm of WO_3_(B) is classified as characteristic type III according to the IUPAC nomenclature and the BET surface area is 13 m^2^ g^−1^, indicating a nonporous structure. The isotherm of CaWO_4_@SiO_2_ is classified as characteristic type II and showed a slight increase in N_2_ uptake at a relative pressure (*P*/*P*_0_) below 0.1, indicating the existence of micropores in the silica shell. The BET surface area of CaWO_4_@SiO_2_ was found to be 65 m^2^ g^−1^. The isotherm of WO_3_@SiO_2_ showed a characteristic type IV with a hysteresis loop and the BET surface area was 175 m^2^ g^−1^. Compared with CaWO_4_@SiO_2_, WO_3_@SiO_2_ showed a steep increase in N_2_ uptake at *P*/*P*_0_ below 0.1, indicating the formation of a well-developed micropore system probably due to removal of the surfactant in the silica shell during high temperature calcination. Moreover, a hysteresis loop enclosed at a *P*/*P*_0_ of *ca.* 0.4–0.9 was observed. These phenomena are attributed to the tensile strength effect observed in materials having a hollow structure encapsulated by a pore system of relatively small pore size.^32^ These results are in agreement with the TEM images and indicate the formation of a hollow structure with a well-developed pore system in the silica shell.

The content of the WO_3_ component in WO_3_@SiO_2_ was estimated by the gravimetric method. The weight of the WO_3_ component in WO_3_@SiO_2_ was measured after etching the SiO_2_ component by diluted HF aqueous solution; we confirmed in advance that WO_3_ was not dissolved in the dilute HF aqueous solution. From the measurement, the WO_3_ content was found to be 60 wt% of WO_3_@SiO_2_.

**Fig. 4:**
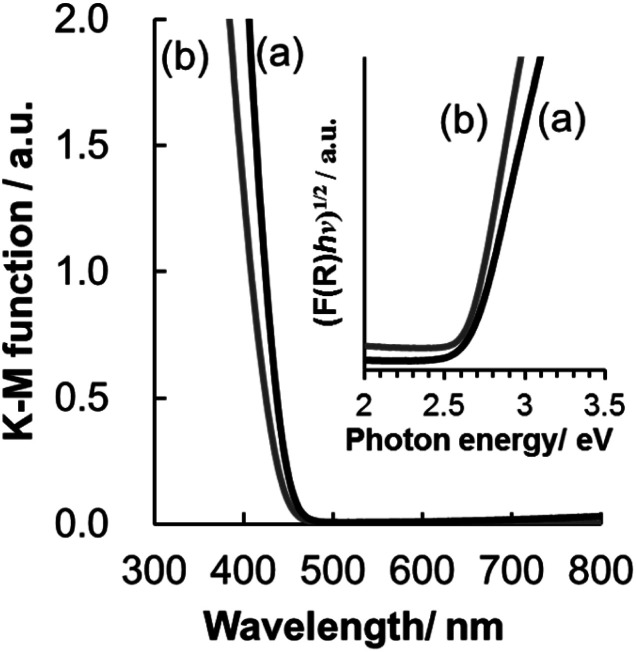
UV-visible diffuse reflectance spectrum of (a) WO_3_@SiO_2_ and (b) WO_3_(B).

UV/Vis diffuse reflectance spectra of WO_3_@SiO_2_ and WO_3_(B) powders are shown in Fig. 4. Reflectance is converted to absorbance by the Kubelka–Munk equation:

1 *F*(*R*) = (1 − *R*)^2^/2*R*,

where *F*(*R*) denotes Kubelka–Munk function and *R* denotes reflectance of the sample relative to that of a non-absorbing standard (BaSO_4_). The spectra indicate that the on-set values of the absorption bands are located at 454 and 486 nm for WO_3_@SiO_2_ and WO_3_(B), respectively. Considering that WO_3_ is an indirect n-type semiconductor, its energy band gap (*E*_g_) can be determined by optical absorption measurements using the following relationship:^33^

2 (*F*(*R*)*hν*)^1/2^ = *K*(*hν* − *E*_g_),

where *hν* the incident photon energy and *K* is a constant. The inset in Fig. 4 shows plots of (*F*(*R*)*hν*)^1/2^*versus hν*. By extrapolating the plots linearly, the energy band gaps of WO_3_@SiO_2_ and WO_3_(B) are estimated to be 2.65 and 2.63 eV, respectively. The estimated energy band gaps are in good agreement with the values (2.5–2.8 eV) reported in the literature.^34^ The reflectance spectrum of the WO_3_@SiO_2_ powder showed a slight blue shift compared with that of the bulk WO_3_(B) powder, probably due to the differences of WO_3_ particle size and/or crystallinity.^35,36^

### Photocatalytic decomposition of acetic acid

Photocatalytic decomposition of acetic acid in gaseous phase was used as a measure of photocatalytic activity of WO_3_ having different morphologies. Since WO_3_ photocatalysts show poor catalytic activity for decomposition of organic compounds due to their low reduction property, we loaded a Pt co-catalyst on WO_3_ particles of all of the photocatalysts we examined; these particles are denoted by Pt/WO_3_.

**Fig. 5:**
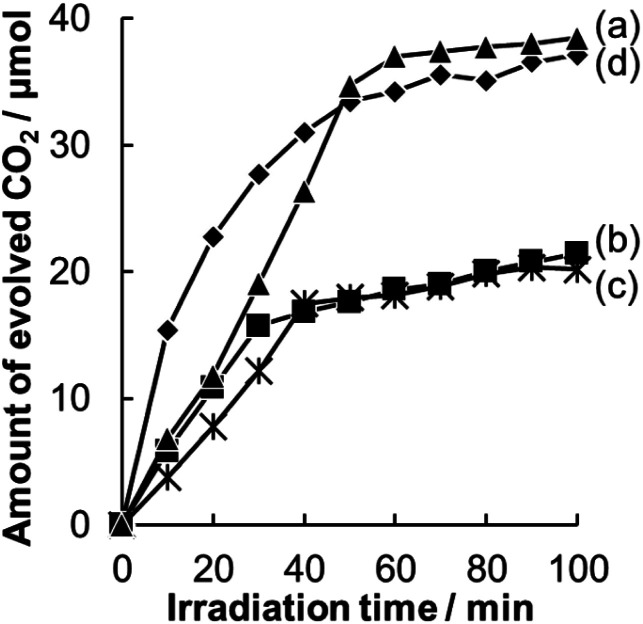
Time courses for CO_2_ evolution during photocatalytic decomposition of acetic acid over (a) Pt/WO_3_@SiO_2_, (b) Pt/WO_3_(B), (c) Pt/WO_3_(B)/SiO_2_ and (d) TiO_2_ (ST-01) under full-arc light irradiation from a 300 W Xe lamp.

**Fig. 6:**
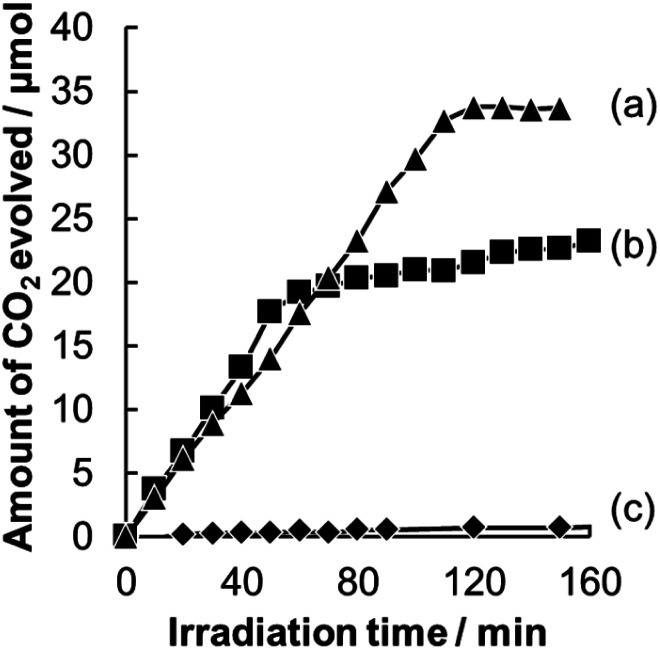
Time courses for CO_2_ evolution during photocatalytic decomposition of acetic acid over (a) Pt/WO_3_@SiO_2_, (b) Pt/WO_3_(B) and (c) TiO_2_ (ST-01) under visible light irradiation (*λ* > 400 nm).

A comparison of time courses of CO_2_ evolution during decomposition of acetic acid under full-arc irradiation from a 300 W Xe lamp with various Pt-loaded WO_3_ photocatalysts and TiO_2_ photocatalyst made of commercially available TiO_2_ (ST-01, Ishihara Sangyo Co. Ltd.) is shown in Fig. 5. In 100 min, the TiO_2_ and Pt/WO_3_@SiO_2_ photocatalysts produced CO_2_ continuously to the level of 35 μmol, which corresponds to the stoichiometric amount of the introduced acetic acid (17.5 μmol), indicating complete decomposition of acetic acid. The initial high rate of CO_2_ evolution with the TiO_2_ photocatalyst seems to be due to its intrinsically high photocatalytic activity. However, the WO_3_ photocatalyts (Pt/WO_3_@SiO_2_ and Pt/WO_3_(B)) showed much higher activity than that of the TiO_2_ photocatalyst, as shown in Fig. 6, suggesting the usefulness of WO_3_ photocatalysts under visible light conditions. Of the WO_3_ photocatalyts, Pt/WO_3_@SiO_2_ was effective for complete decomposition of acetic acid, as shown in Fig. 5 and 6.

In Fig. 5, Pt/WO_3_@SiO_2_ and Pt/WO_3_(B)/SiO_2_ show concave slopes for CO_2_ evolution in the initial period. This unique property of the photocatalyts seems to be related to the hollow shell structure. We consider that the release of CO_2_ from the hollow space of the photocatalysts to the outer space is hindered to some extent by the porous SiO_2_ shell, leading to the concave slope until the concentration of CO_2_ reaches a certain level. For the same reason, we can expect that reactive products produced in the hollow space have as higher chance of reaction on the photocatalyst because their escape from the hollow space and supply of acetic acid to the hollow space are hindered by the silica shell. The concentration of the intermediate in the hollow shell of Pt/WO_3_@SiO_2_ can be lower than that of Pt/WO_3_(B) in the gas phase because the intermediate competes with acetic acid for the reaction sites (or photogenerated holes) on WO_3_. Such a function of the hollow shell (or hollow shell effect) will affect the activity of the photocatalyts as mentioned later.

Pt/WO_3_@SiO_2_ and Pt/WO_3_(B) showed almost the same reaction rates for CO_2_ evolution during the initial reaction period, as shown in Fig. 5. However, the evolution rate of CO_2_ with Pt/WO_3_(B) decreased drastically when the amount of CO_2_ reached half of the stoichiometric level, suggesting that methanol or formaldehyde, both of which are the chief intermediates for decomposition of acetic acid, accumulates in the system. To confirm this assumption, we added acetic acid to the system with Pt/WO_3_(B) after the CO_2_ evolution rate had decreased and we found that the CO_2_ evolution rate almost recovered to the initial rate (Fig. S3†). This result strongly suggests that the decrease in the CO_2_ evolution rate with Pt/WO_3_(B) is not due to poisoning of the active sites of WO_3_ but is due to the slow decomposition rate of the intermediate. This is also consistent with the fact that CO_2_ evolution continued even after the CO_2_ evolution rate had decreased over Pt/WO_3_(B).

To confirm the effect of the intermediates, products in the gas phase were analyzed during the photocatalytic reaction. As a result, methanol was observed in the gas phase together with very small amounts of methyl formate and methyl acetate. However, formaldehyde was not observed. It is well known that formaldehyde is the major oxidation product from methanol.^37^ Formaldehyde was not detected in this system probably because formaldehyde was strongly adsorbed on the photocatalysts or decomposed rapidly. The amount of methanol over Pt/WO_3_@SiO_2_ was smaller than that over Pt/WO_3_(B) (Fig. S4†). This result is consistent with the hollow shell effect mentioned above.

In the case of Pt/WO_3_(B), even after methanol and other intermediates had been completely decomposed, the amount of CO_2_ was insufficient for a stoichiometric amount, indicating that the formaldehyde produced is strongly adsorbed on Pt/WO_3_(B). Formaldehyde is easily polymerized to a stable paraformaldehyde at a high concentration as reported in the literature.^38,39^ Since paraformaldehydes are relatively difficult to decompose, the CO_2_ evolution rate is drastically reduced if they accumulate on the photocatalyst.

In the case of the hollow space of Pt/WO_3_@SiO_2_, however, the sequential photo decomposition reaction of intermediates proceeds efficiently due to the hollow shell effect, which maintains the concentration of formaldehyde at a low level. As a result, formation of paraformaldehyde was probably suppressed and continuous complete decomposition of acetic acid was achieved. The catalytic activity of Pt/WO_3_(B)/SiO_2_, which is physically mixed WO_3_(B) with SiO_2_, was almost the same as that of Pt/WO_3_(B). These results indicate that the hollow structure, not the SiO_2_ content, is important for the complete decomposition of acetic acid. Although further studies are needed to understand the detailed reaction mechanisms, the hollow structure is expected to be effective for the photodecomposition of organic compounds through stable intermediates.

## Conclusions

We developed a procedure for the synthesis of WO_3_ nanoparticles encapsulated in a hollow silica sphere by effectively using a reverse micelle system and volume change of tungsten compounds. The obtained core–hollow shell structure was composed of WO_3_ nanoparticles of 10 nm in diameter and a porous SiO_2_ shell of 10 nm in thickness with highly developed micropores. The core–hollow shell structure of Pt-loaded WO_3_@SiO_2_ has great advantages in photocatalytic applications, as exemplified by the continuous and complete decomposition of acetic acid, compared to other bulk WO_3_ photocatalyts. The findings will lead to further improvement in the catalytic activity of WO_3_@SiO_2_ by an optimized core–hollow shell structure.

## Conflicts of interest

There are no conflicts to declare.

## Supplementary Material

RA-010-D0RA01988G-s001
