# Supplementary material for: Synthesis of nano-sized tungsten oxide particles encapsulated in a hollow silica sphere and their photocatalytic properties for decomposition of acetic acid using Pt as a co-catalyst
Source: RSC Adv. 2020 Apr 17;10(26):15360–5. doi: 10.1039/d0ra01988g (PMC9052306; doi:10.1039/d0ra01988g)
Supplement: RA-010-D0RA01988G-s001 [file RA-010-D0RA01988G-s001.pdf]

Electronic Supplementary Material (ESI) for RSC Advances.
This journal is © The Royal Society of Chemistry 2020

Synthesis of nano-sized tungsten oxide particles encapsulated in a hollow silica sphere and their photocatalytic properties for decomposition of acetic acid using Pt as co-catalyst

T. Harada[1,*], E. Yagi[1], S. Ikeda[2]

[1]*Research Center for Solar Energy Chemistry, Osaka University, 1-3 Machikaneyama, Toyonaka 560-8531, Japan*

[2]*Department of Chemistry, Faculty of Science and Engineering, Konan University, Okamoto, Higashinada, Kobe 658-8501, Japan.*

* Corresponding Author: Takashi HARADA

E-mail address: harada@chem.es.osaka-u.ac.jp

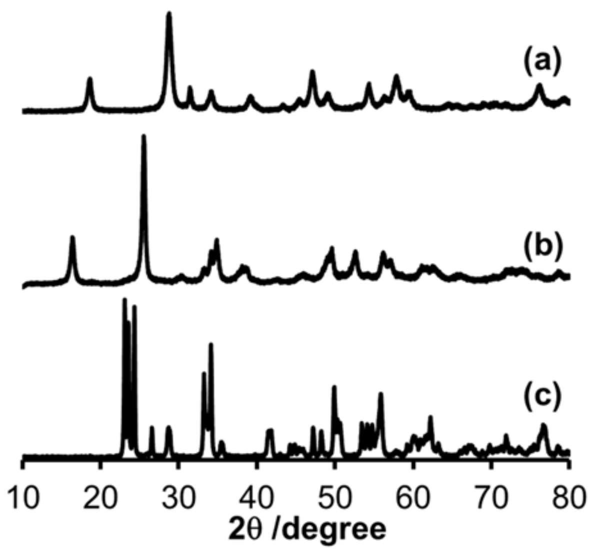

Fig. S1 XRD patterns of (a) $CaWO_4$, (b) H_2WO_4 and (c) WO_3.

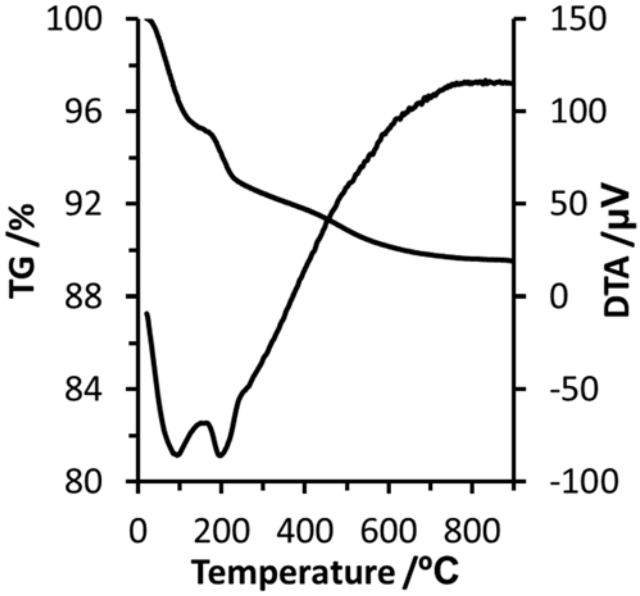

Fig.S2 TG–DTA curves of the $H_2WO_4@SiO_2$.

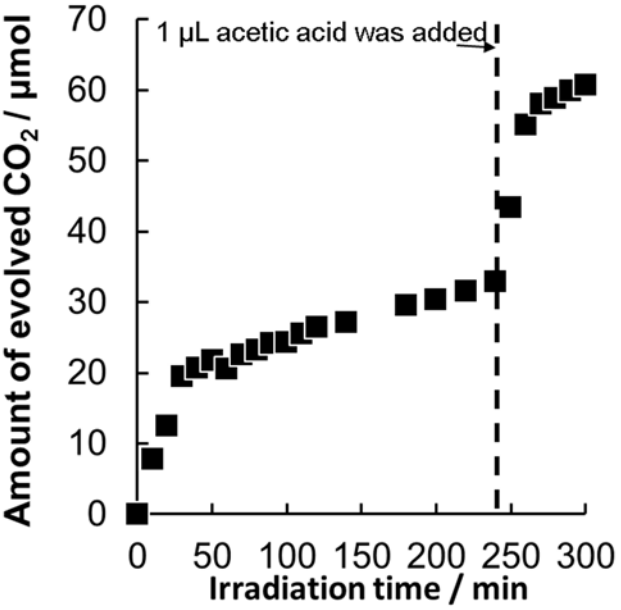

Fig. S3 Time course for CO_2 evolution during photocatalytic decomposition of acetic acid over Pt/WO_3(B) under full-arc light irradiation. One μl of acetic acid was added into the reactor at 240 minutes.

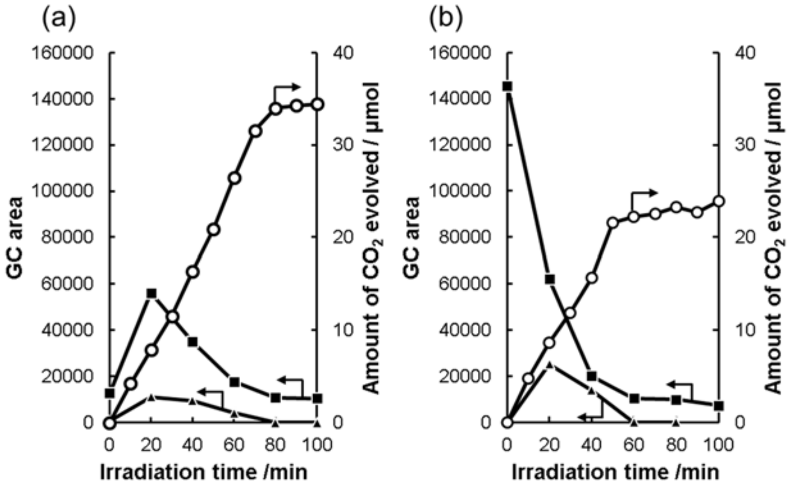

Fig. S4 Time courses of CO_2 evolution, acetic acid decomposition and methanol production during photocatalytic decomposition of acetic acid over (a) Pt/WO_3@SiO_2 and (b) Pt/WO_3(B) under full-arc light irradiation. For the analysis of the intermediates, the reaction was carried out under a weak light intensity condition.
